# Spontaneous Cystogastrostomy: A Natural Response

**DOI:** 10.7759/cureus.27250

**Published:** 2022-07-25

**Authors:** Sara Izwan, Erick Chan, Ramesh Damodaran Prabha, Harald Puhalla

**Affiliations:** 1 General Surgery, Gold Coast University Hospital, Gold Coast, AUS; 2 School of Medicine and Dentistry, Griffith University, Gold Coast, AUS; 3 Faculty of Health Sciences and Medicine, Bond University, Gold Coast, AUS

**Keywords:** pseudocyst drainage, hepato-biliary-pancreatic surgery, internal pancreatic fistula, cystogastrostomy, alcoholic pancreatitis

## Abstract

Pancreatic pseudocysts are a common complication of pancreatitis. Conservative management and repeat imaging are appropriate to monitor spontaneous regression. However, in some cases, rupture and haemorrhage of pseudocysts can lead to life-threatening events requiring urgent intervention. We present a male patient in his 30s who was presented to the emergency department with severe pancreatitis in the context of alcohol excess. Past medical history included pancreatitis with a small pseudocyst and splenic vein thrombosis for which he was anticoagulated six weeks previously. Computer tomography of the abdomen and pelvis showed an interval increase in his pseudocyst with haemorrhage secondary to a suspected splenic artery pseudoaneurysm. He was admitted for attempted embolisation and observation. Serial imaging demonstrated progression of the pancreatic pseudocyst and then spontaneous interval decompression via a transgastric fistula, leading to a natural cystogastrostomy confirmed on subsequent endoscopy. We discuss the uncommon sequelae of a complication of pancreatitis, and consider the hypotheses related to this rare occurrence, with suggestions for management and follow-up of these patients.

## Introduction

Pancreatic pseudocysts are a common complication of pancreatitis. The incidence of pancreatic pseudocysts is about 5% to 15% in acute pancreatitis [1-3], 20% to 40% in chronic pancreatitis [1], and is most commonly seen in alcoholic chronic pancreatitis in 70% to 78% [2]. A pancreatic pseudocyst is defined according to the revised Atlanta classification as an encapsulated collection of fluid with a well-defined inflammatory wall with minimal or no necrosis that develops with a delay of at least four weeks to the initiating event [4]. The main mechanism that leads to the formation of pancreatic pseudocysts is disruption of the main pancreatic duct and/or peripheral ductules, leading to leakage and extravasation of pancreatic enzymes, which causes localised autodigestion and necrosis of the pancreatic parenchyma [5]. This leads to a localised inflammatory response with the formation of a distinct wall composed of granulation tissue and vessels that organises with more connective tissue and fibrosis [5,6].

Patients often present with abdominal pain, nausea, and vomiting due to compression of the pseudocysts on surrounding intra-abdominal organs, and a diagnosis is made with confirmation via imaging. Management is usually conservative, including a period of observation to allow the maturation of the cystic wall, and in up to 25% of cases, acute pseudocysts will undergo spontaneous resolution [3]. When surgical or endoscopic intervention is indicated, care must be taken to consider the presence and risk of local complications. Endoscopic cystogastrostomy is commonly used to drain the pseudocyst and alleviate some of the mass effect on surrounding viscera. Otherwise, resolution of a pseudocyst can occur when proteolytic action of the pancreatic enzymes causes erosion of the pseudocyst wall and a fistula is formed between the surrounding colon or small bowel, which occurs in less than 3% of cases reported in the literature [7,8]. Spontaneous fistulisation into the stomach is an extremely rare phenomenon. We postulate the reason for this rare occurrence is due to the stomach being a very pliable, thick-walled organ, and therefore, less prone to pressure necrosis compared to surrounding viscera.

We present a case report of a young male who presented with two unusual complications of his acute pancreatitis; a haemorrhagic transformation of the pseudocyst in the absence of a clearly identified arterial pseudoaneurysm, and a spontaneous fistulisation of the same into the stomach. Ethics was obtained prior to the commencement of this report. Informed consent was obtained from the patient for the preparation of this report.

## Case presentation

A young male in his early 30s presented to the emergency department (ED) with a three-day history of epigastric pain, nausea, and vomiting. His medical history was significant for alcohol-induced pancreatitis six weeks previously, and he had been discharged after a recent admission for the same. His initial presentation was complicated by necrosis of the pancreatic tail and splenic vein thrombosis, for which he was commenced on therapeutic anticoagulation. He was discharged on day 11 of his initial presentation, with interval imaging suggestive of complete resolution of his splenic vein thrombosis. He presented to the ED six weeks later with ongoing abdominal pain and fever. On examination, he was pale and tachycardic, with mild tenderness to the epigastrium. Computer tomography (CT) imaging was suggestive of a likely haemorrhagic pancreatic pseudocyst from a suspected splenic artery pseudoaneurysm, in the context of his anticoagulation. His presentation was further complicated by a positive COVID-19 polymerase chain reaction (PCR) result, as a reversal of anticoagulation was deemed to further increase his thrombosis risk. He was responsive to resuscitation in the ED and was admitted to the intensive care unit for close observation. No arterial source of bleeding was identified for embolisation. An endoscopic cystogastrostomy was attempted and aborted when he was noted to have a large transgastric fistula leading to natural drainage of his pseudocyst.

Investigations

**Figure 1:**
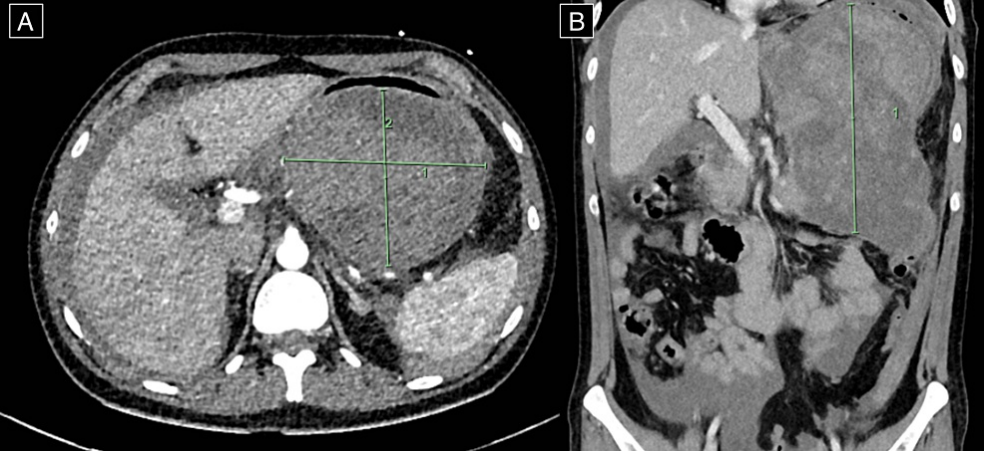
(A) Axial view of computer tomography (CT) of the abdomen showing a large encapsulated collection suggestive of walled-off necrosis measuring 105 × 120 × 178 mm posterior to the stomach. (B) Coronal view demonstrating the length of collection measuring 178 mm.

Pathology and imaging results were available to the surgical team at the time of referral. Laboratory examinations showed macrocytic anaemia, with haemoglobin on the arrival of 54 g/L (reference range 135-180) and leukocytosis of 17.2 × 10^9^/L (reference range 4-11). The lipase was elevated at 1390 units/L (normal high <60). Abdominal CT demonstrated a large, encapsulated collection posterior to the stomach associated with the anterior border of the pancreatic tail consistent with walled-off necrosis measuring 105 × 120 × 178 mm (Figure 1) with compression onto adjacent viscera.

**Figure 2:**
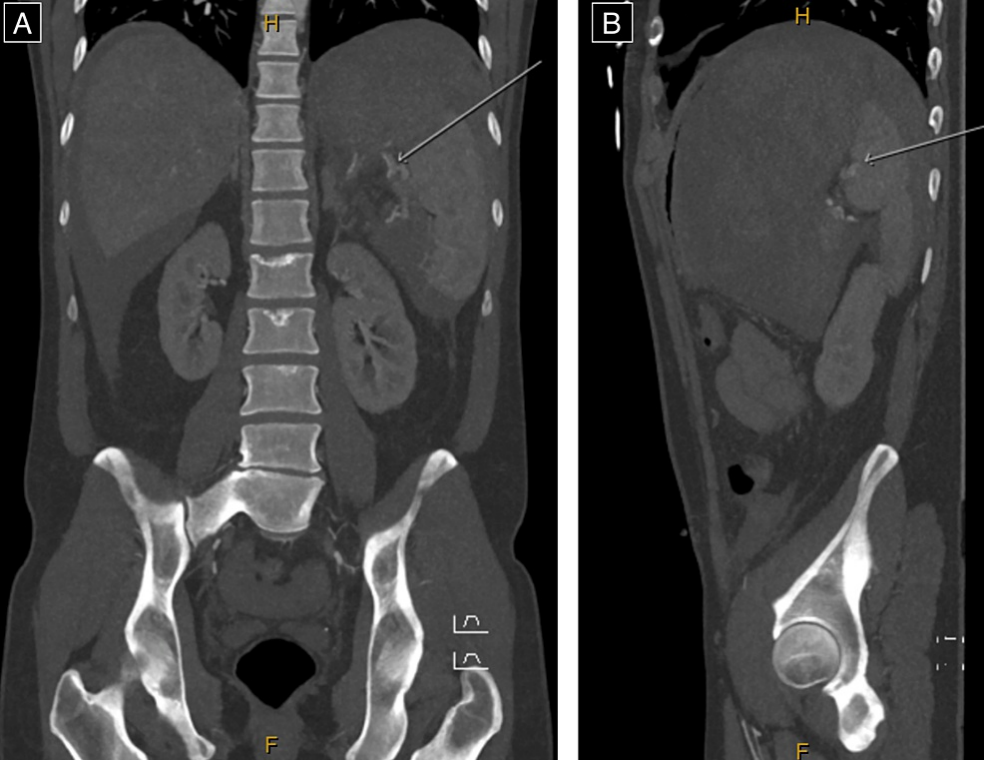
CT angiogram images demonstrating possible small splenic artery pseudoaneurysm (note arrows) at the splenic hilum on (A) coronal and (B) sagittal sections. CT: computer tomography.

CT angiogram demonstrated a possible small splenic artery pseudoaneurysm, with an early filling of the splenic vein concerning for an arterio-venous fistula (Figure 2). 

**Figure 3:**
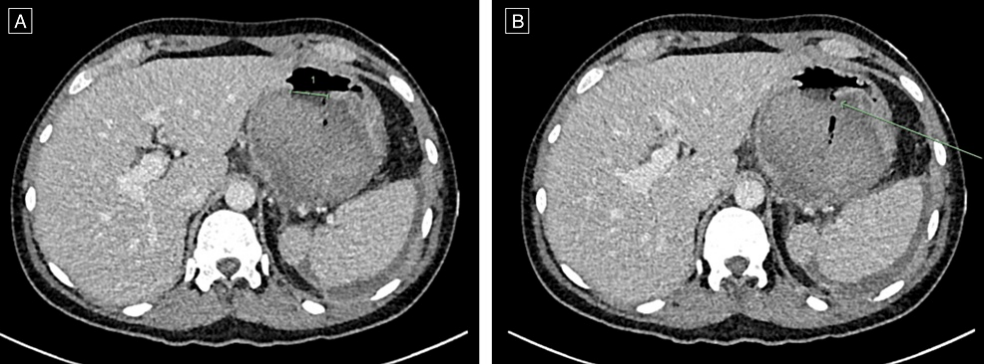
CT abdomen demonstrating interval decompression of the large pancreatic pseudocyst. (A) A new 25 mm defect is demonstrated involving the posterior wall of the stomach. (B) Internal gas locules within the lobulated pseudocyst are in keeping with a contained perforation and subsequent infected pseudocyst. CT: computer tomography.

Embolisation was attempted; however, no target vessel was identified. Interval CT imaging two weeks later demonstrated interval decompression of the pancreatic pseudocyst due to a spontaneous fistulisation and drainage to the stomach (Figure 3). 

Treatment

**Figure 4:**
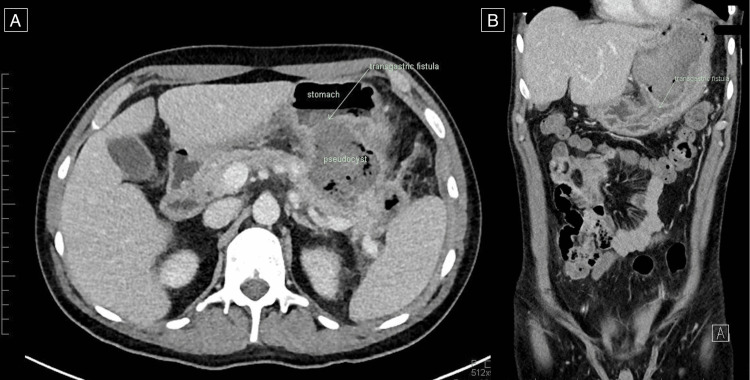
CT abdomen demonstrating interval decompression of the large pancreatic pseudocyst. A new defect is demonstrated involving the posterior wall of the stomach, where a large transgastric fistula between the pancreatic pseudocyst and the posterior wall of the stomach (A) in axial view and (B) in coronal view. CT: computer tomography.

The patient underwent an upper gastrointestinal gastroscopy and endoscopic ultrasound, which confirmed a large, >3 cm spontaneous transgastric fistula from the pancreatic pseudocyst into the proximal stomach (Figure 4).

**Figure 5:**
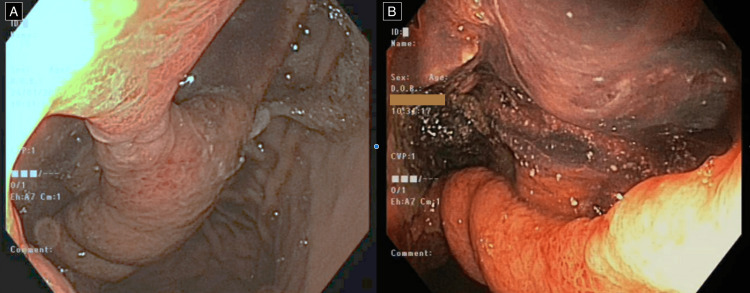
Endoscopic images at the time of endoscopic retrograde cholangiopancreatography (ERCP) demonstrating (A) the large transgastric fistula >3 cm with a small tissue bridge in the middle and (B) a haemorrhagic necrosum inside the pseudocyst cavity.

Necrosectomy was not performed given biochemical and clinical improvement. Endoscopic images (Figure 5) are provided, demonstrating the transgastric fistula appreciable at the time of endoscopic retrograde cholangiopancreatography (ERCP).

Outcome

The patient was discharged once his symptoms improved. At six weeks post-discharge, he was able to return to work with no complaints of pain or further complications. An interval CT of the pancreas demonstrated resolution of the large pancreatic pseudocyst with some moderate residual bulkiness of the tail of the pancreas due to previous pancreatic necrosis. He was reviewed in the hepatobiliary outpatient clinic, where he admits to still drinking alcohol regularly. Alcohol cessation advice was reiterated, as well as encouragement to continue to engage regularly with his general practitioner in the community.

## Discussion

Pancreatic pseudocysts are a common complication of pancreatitis, with an incidence of between 5% to 15% in acute pancreatitis [1-3] and 20% to 40% in chronic pancreatitis [1]. Their aetiologies are most commonly biliary (75.4%), alcoholic (10.3%), secondary to surgery (3.9%), idiopathic (2%), or trauma-related (8.4%) [9]. Although pseudocysts themselves are not rare, spontaneous perforation or fistulisation into surrounding organs accounts for less than 3% of these cases, which may be associated with life-threatening haemorrhage requiring emergency intervention [7].

In uncomplicated cases, regression of a pseudocyst occurs by natural drainage to the duodenum through the pancreatic duct. When a fistula is formed, leakage of rupture allows drainage from the pseudocyst into the gastrointestinal tract. The pathogenesis behind the bleeding and rupture of pancreatic pseudocysts has been suggested. Firstly, acute inflammation from activated lytic enzymes such as elastase and trypsin likely erodes the cyst wall through progressive digestive processes [5]. This is similar in the pathophysiology of pancreatic pseudoaneurysms, through erosion of elastin in vessel walls, which have a high risk of rupture and haemorrhage. Secondly, large pseudocysts exert a mass effect on surrounding vessels and cause erosion from pressure necrosis in addition to enzymatic contact [8]. In the third instance, inflammation from the pseudocyst may cause compression or thrombosis in the portal or splenic vein, leading to portal hypertension [7,10].

Several cases have been reported of spontaneous pancreatic pseudocystocolonic [11], pancreaticopleural [12], and cutaneous [13] fistulisation of the pseudocyst into the lumbar region. There are currently only a few cases in the literature of spontaneous rupture of pancreatic pseudocysts into the stomach due to natural fistulisation, leading to subsequent resolution of the pseudocyst without the need for endoscopic or surgical intervention [7,10,14-17].

Pancreatic pseudocysts have been treated surgically for over 40 years. Now, newer techniques are steadily becoming more common, such as internal drainage via cystogastrostomy and cystojejunostomy. There is no current consensus as to the gold standard for the treatment of pseudocysts, but a study by Pan et al. has created a classification system based on anatomical location and clinical manifestation of the pseudocyst and recommendations for treatment [9]. They recommended consideration of endoscopic drainage for any pseudocyst cavity, with comparable success rates and outcomes between surgical and endoscopic groups [9]. In this case presentation, our patient had a suspected natural transgastric fistula, which was confirmed endoscopically on attempted drainage. His presentation was complicated by anaemia secondary to the suspected haemorrhagic transformation of his pseudocyst. Interestingly, with no clear pseudoaneurysm appreciated on attempted angiographic embolisation, it is likely this patient had a haemorrhagic pseudocyst from a venous source in the context of his anticoagulation, which then self-resolved. In this case, as this patient remained haemodynamically stable, it was reasonable to continue non-operative management with close observation and serial imaging.

## Conclusions

Pancreatic pseudocysts are a common complication of recurrent acute or chronic pancreatitis. Management often involves endoscopic, surgical, or percutaneous options for drainage to alleviate symptoms associated with large, complex pseudocysts. Natural fistulisation of the pancreatic pseudocyst into the stomach is a rare occurrence that achieves the same effect as an endoscopic cystogastrostomy, which is relevant in this patient’s case. Consideration must be given to best practice management of patients who are also concurrently positive for COVID-19.
